# Effect of Chitin Nanofibrils on Biocompatibility and Bioactivity of the Chitosan-Based Composite Film Matrix Intended for Tissue Engineering

**DOI:** 10.3390/ma12111874

**Published:** 2019-06-10

**Authors:** Natalia V. Smirnova, Konstantin A. Kolbe, Elena N. Dresvyanina, Sergey F. Grebennikov, Irina P. Dobrovolskaya, Vladimir E. Yudin, Thomas Luxbacher, Pierfrancesco Morganti

**Affiliations:** 1 Institute of Macromolecular Compounds, Russian Academy of Sciences, V.O. Bolshoi pr. 31, 199004 St. Petersburg, Russia; kkolbe@yandex.ru (K.A.K.); dobrov@hq.macro.ru (I.P.D.); yudin@hq.macro.ru (V.E.Y.); 2 Saint-Petersburg State University of Industrial Technologies and Design, ul. Bolšaja Morskaja 18, 191183 St. Petersburg, Russia; elenadresvyanina@gmail.com (E.N.D.); grebennikovsf@gmail.com (S.F.G.); 3 Anton Paar GmbH, Anton-Paar-Str. 20, 8054 Graz, Austria; thomas.luxbacher@anton-paar.com; 4 Department of Experimental Medicine, University of Campania “Luigi Vanvitelli”, Via L. De Crecchio 7, 80138 Naples, Italy; pierfrancesco.morganti@mavicosmetics.it

**Keywords:** chitosan, chitin nanofibrils, composite matrix, tissue engineering

## Abstract

This paper discusses the mechanical and physicochemical properties of film matrices based on chitosan, as well as the possibility of optimizing these properties by adding chitin nanofibrils. It is shown that with the introduction of chitin nanofibrils as a filler, the mechanical stability of the composite materials increases. By varying the concentration of chitin nanofibrils, it is possible to obtain a spectrum of samples with different bioactive properties for the growth of human dermal fibroblasts. Film matrices based on the nanocomposite of chitosan and 5 wt % chitin nanofibrils have an optimal balance of mechanical and physicochemical properties and bioactivity in relation to the culture of human dermal fibroblasts.

## 1. Introduction

Although significant advancements have already been made in the development of biomaterials, regenerative technologies still demand polymer materials that can be processed without harming the environment, are biocompatible, biologically active, and capable of safe bioresorption. The biopolymers chitin (CN) and chitosan (CS) completely meet the above requirements; in addition to these properties, they have some other advantages, such as antibacterial and fungicidal activity, anesthetic, wound healing, and hemostatic properties [1].

Two important physicochemical parameters of CS are its degree of deacetylation and its molecular mass. These characteristics have a significant influence on the properties of CS-based materials and, therefore, on their biomedical applications [2]. Use of high molecular mass chitosan is restricted in a number of areas since it has low solubility in water and diluted solutions of acids; besides, its solutions demonstrate high viscosities even at low concentrations. Low molecular mass chitosan samples have biomedical applications due to their high solubility in water and low viscosity of aqueous solutions at physiological pH values [3].

The degree of deacetylation has an effect on biocompatibility, biodegradability, solubility in acidic solutions, hydrophilicity, swelling in water, and, therefore, on the biological activity of CS [4]. Since the matrices intended for various tissue regeneration techniques require different degradation rates, the possibility of controlling the chitosan bioresorption process makes this polymer applicable for the regeneration of almost all types of tissues.

The cationic nature of CS favors the formation of polyelectrolyte complexes with a wide range of anionic glycosaminoglycans, including heparin, heparin sulfate, and chondroitin sulfate. Glycosaminoglycans, especially heparin and heparin sulfate, are able to bind growth factors and cytokines, as well as initiate or induce their synthesis. Thus, the constructions containing chitosan-glycosaminoglycan complexes can be used for retaining and accumulating the necessary factors that are released by colonizing cells and for accumulating growth factors from the surrounding biological fluids. The important role of these complexes in regeneration technologies for bone and cartilage tissues has been demonstrated [5].

The presence of functional groups in CS provides an opportunity to obtain conjugates between chitosan and biologically active molecules, such as laminin peptides, RGD-peptides/proteins, γ-poly(glutamic acid), etc. Thus, the matrix can be used for immobilization of biologically active molecules that participate in the adhesion, proliferation, differentiation of cells, and other processes that are important for tissue regeneration [6].

Studies of physicochemical and mechanical properties of CS made it possible to develop numerous techniques for processing, modifying, and preparing different types of chitosan-based matrices intended for regenerative medicine. A wide selection of matrices (fibers, films, sponges, and hydrogels) provides a means to simulate surface properties and mechanical characteristics of a natural tissue.

Despite all the advantages described above, chitosan has several limitations, such as the absence of biological response, which is partially caused by mechanical properties of CS (inappropriate for many applications) and strong swelling of CS in liquid media. Besides, difficulties in the use of pure CS as a basis for cell cultivation matrices may be related to electrostatic interactions between positively charged amino groups of CS and the cell surface. This interaction disturbs the activity of trans-membrane cell receptors. This disturbance, in turn, has an adverse effect on cell-surface adhesion and cell proliferation [7]. A decrease of surface charge helps to reduce this effect.

With this, one method for optimizing properties of CS-containing matrices is introducing mineral or organic nano-sized fillers. Successful use of the composites strongly depends on high specific area of nanoparticles, which provides a high surface-area-to-volume ratio and, therefore, stronger interaction between the polymer and the nanofiller. Besides, during stable interphase interaction, excellent properties of nanoparticles are transferred to the composite [8]. Preparation of nanocomposites allows varying properties of natural polymers, including chitosan (e.g., their porosity, surface morphology, and mechanical strength), over a wide range, and imparts new functional properties to native macromolecules [9].

A variety of biomedical technologies require completely bioresorbable materials (i.e., containing bioresorbable matrix and filler). One of the biocompatible, bioactive, and bioresorbable polymers is chitin, which can be successfully used as a filler. The main structural units of chitin are nanofibrils; they are highly oriented macromolecular aggregates with transverse sizes of 15–20 nm and longitudinal sizes of 400–500 nm [10]. This structure provides a high mechanical strength of natural chitin-containing tissues. The crystalline structure of chitin is polymorph and consists of two crystalline modifications with an antiparallel (α-chitin) or a parallel (β-chitin) arrangement of macromolecules.

A number of biomedical studies have demonstrated the possibility of modifying chitosan and other polymers by introducing a filler consisting of chitin nanofibrils (chitin nanowhiskers, CNWs) [11]. In vitro studies show that CNWs (which possess a high surface area) effectively interact with enzymes, proteins, and cell components of blood, immune cells, fibroblasts, and other cells involved in tissue regeneration. Thus, when CNWs are used in regenerative technologies, modulation of granulation processes takes place, angiogenesis and regular deposition of collagen fibers in the damaged areas are stimulated, and re-epithelialization proceeds more effectively [12]. At the same time, the antiseptic action of CNWs plays an important role in the healing process; besides, their presence reduces the risks of scar tissue hypertrophy and contraction [13].

However, any reinforcement agent not only changes the mechanical behavior of the basic material, but it might also influence its biological performance, such as its biocompatibility, biodegradability, or bioactivity. In the best case, the contribution of a reinforcing compound is beneficial and leads to an improvement of the biological performance of a composite. To evaluate the influence of the structure, chemical composition, and content of a reinforcing agent on biological properties of a composite, a generic approach should be used. An ideal system for such investigations should be readily producible, form homogeneous composite structures, and allow for an easy modification of a reinforcing agent. It should be possible to use a wide range of physicochemical, mechanical, and biological methods to characterize the prepared materials. Furthermore, the interaction between a biomaterial’s surface and biological matter is a crucial aspect in the design of biomaterials and a critical factor for their performance. Protein adsorption is important since the initial protein layer mediates cell colonization and directs the overall biocompatibility. The initial studies of a biomaterial’s surface should include the determination of general surface properties (chemical composition, mechanical properties, degree of hygroscopicity, surface charge) and the investigation of its interactions with different kinds of cells.

The first aim of the present study was to obtain film matrices that possess the mechanical properties necessary for sterilization, cell cultivation, and other manipulations in liquid media. For this purpose, we studied the effect of chitin nanofibril additives on mechanical properties, moisture sorption, and surface charge of chitosan films. Furthermore, analysis of proliferation characteristics of the cells cultivated on modified substrates allowed us to find the optimal composition of the nanocomposites. The investigation of these relationships will help to optimize biocompatibility and bioactivity of the advanced matrices for tissue engineering.

## 2. Materials and Methods

### 2.1. Preparation of Composite Films

Chitosan (Biolog Heppe GmbH, Landsberg, Germany) and chitin nanofibrils (Mavi Sud srl, Aprilia, Italy) were used for preparing composite films. The molecular mass (M_m_) of chitosan was 1.64 × 10^5^ g/mol and the degree of deacetylation (DD) was 92%. The diameter of chitin nanofibrils was 20 ± 4 nm and the length was 700 ± 100 nm [10,14]. For evaluating the effect of the film matrix on the properties of the composite films, chitosan with a similar M_m_ of 1.4 × 10^5^ g/mol but a lower DD of 80% was used (Sigma Aldrich, St. Louis, MO, USA). Glacial acetic acid (Vecton ZAO, Moscow, Russia) was used as a solvent.

Composite films were prepared from aqueous acetic acid solutions of chitosan containing chitin nanofibrils. In an amount necessary to produce a solution with a chitosan concentration of 4 wt %, chitosan was added to the aqueous dispersion of chitin nanofibrils. The content of chitin nanofibrils with respect to chitosan was 0, 0.5, 1, 5, 10, 20, 30 wt %. Before the addition of chitosan, a suspension containing chitin nanofibrils was dispersed to uniformly distribute particles throughout the volume. This was done using an ultrasonic generator IL10-0.63 for 15 min at a frequency of 23 kHz. The resulting solution was stirred for 30 min at 1000 rpm, after which acetic acid was added in such an amount that its concentration in the solution was 2%. The resulting solution was mixed for 60 min at 1000 rpm. After that, the chitosan solution with chitin nanofibrils was filtered and deaerated at a low pressure of 0.8 atm for 24 h.

The chitosan and composite films were obtained by casting the solution through a slit draw die onto a glass substrate followed by drying at RT (room temperature) for 1 day. The thickness of the films was 80 ± 15 μm. The prepared films were kept in a mixture of 10% aqueous solution of NaOH and C_2_H_5_OH for 15 min, then washed with distilled water, and air-dried.

### 2.2. Mechanical Testing of Materials

The measurements of the mechanical properties of the films were carried out with the electrodynamic system ElectroPuls E1000 (Instron, Norwood, MA, USA) in the mode of stretching the sample in the form of strips 2 mm wide and 20 mm long. Tensile tests were carried out at a speed of 5 mm/min at room temperature. Prior to the mechanical testing, films were stored at a relative humidity (RH) of 66% for 24 h. Ten measurements were taken at each point.

### 2.3. Moisture Sorption Study

Water vapour sorption was measured by the isopiestic method; the RH in the desiccators was created with saturated salt solutions. The desiccators were thermostated at 25 °C. The samples were kept at each partial water vapour pressure until sorption equilibrium was established, where the equilibrium sorption values were established for 3–7 weeks. Before measurement, the samples were dried at 80 °C to a constant weight [15]; the control over the moisture content was carried out by the weight method.

### 2.4. Water Contact Angle

The water contact angle on chitosan and chitosan-based composite films was determined by the sessile drop method using the drop shape analyzer DSA30 (Krüss GmbH, Hamburg, Germany). Distilled water was used as the test liquid. Five measurements were taken for each film sample.

### 2.5. Zeta Potential Analysis

The surface charge of the chitin-chitosan nanocomposite films was assessed by the zeta potential at the film-water interface. The surface zeta potential was determined from the measurement of the streaming potential using the instrument SurPASS™ 3 (Anton Paar, Graz, Austria). The streaming potential was generated by applying a pressure gradient across a capillary channel, which provokes flow of an aqueous solution and is related to the surface zeta potential by

(1) $$\zeta =\frac{dU_{str}}{d∆p}\cdot \frac{\eta }{\varepsilon \cdot \varepsilon_{0}}\cdot \kappa_{B}$$

where d*U_str_*/dΔ*p* is the streaming potential coupling coefficient, η and ε are the viscosity and dielectric coefficient of water, ε_0_ is the vacuum permittivity, and κ*_B_* is the electric conductivity of the bulk solution.

The chitin–chitosan nanocomposite films were stored in 0.001 mol/l KCl for 24 h prior to the zeta potential analysis. For each film composition, two samples with a size of 20 mm × 10 mm were then mounted on sample holders with the same cross-section using double-sided adhesive tape. The sample holders were inserted in the measuring cell (adjustable gap cell) and the distance between the film surfaces was adjusted to 109 ± 4 µm by monitoring the temporal change of the applied pressure difference. The streaming potential was recorded in the pressure range of 200–500 mbar and the streaming potential coupling coefficient dU_str_/dΔp in Equation (1) was taken as the slope of the linear dependence of streaming potential on pressure. The isoelectric point (iep; the iep coincides with the pH of the aqueous solution where ζ = 0 mV) was determined by a pH scan of the zeta potential in the range of pH 6–9.5. At each pH, the zeta potential was recorded four times. A 0.001 mol/l KCl solution was used as the background electrolyte and the pH was adjusted automatically with 0.05 mol/l KOH. Prior to the streaming potential, the Ohm resistance *R* inside the capillary channel was measured under stagnant (no-flow) conditions. The electric conductance, i.e., the inverse Ohm resistance, is an indicator for the swelling propensity of the chitin–chitosan nanocomposite films. To compensate for the effect of a variation in the electrolyte conductivity of the bulk solution, either due to a shift in the solution temperature or due to an increase in the ionic strength by the addition of 0.05 mol/l KOH, the cell constant of the capillary channel calculated according to

(2) $$\frac{L}{A}=\frac{L}{W\cdot L}=R\cdot \kappa_{B}$$

was evaluated and correlated with the swelling propensity instead of either the Ohm resistance or the conductance. *L*, *A*, *W*, and *H* are the length, cross-section, width, and height of the rectangular flow channel between chitin–chitosan nanocomposite films.

### 2.6. Fibroblast Attachment, Viability, Proliferation, and Morphology

Human dermal fibroblasts obtained from fresh skin biopsy were from the Institute of Cytology, RAS (St. Petersburg, Russia) and were used up to the 10th passage. The cells were cultured in Dulbecco modified Eagle medium (DMEM) supplemented with 1% penicillin, 1% streptomycin, 1% fungizone, 2 mM L-glutamine, and 10% fetal bovine serum (FBS). The cells were incubated at 37 °C in a humidified atmosphere containing 5% CO_2_. For the experiments, fibroblasts from pre-confluent cultures were harvested with 0.25% trypsin/EDTA. Trypsin was neutralized with FBS and the cells were re-suspended in DMEM (all Thermo Fisher Scientific, Waltham, MA, USA).

Studies of the viability and proliferation of the cells in the samples were performed using the tetrazolium bromide (MTT) test. Film matrices were placed in 24-well plates, and culture medium was added. Sterilized silicone rings were placed on top to keep the scaffolds submersed, and 25 × 10^3^ fibroblasts were seeded on top of the scaffolds. The cells were resuspended in culture medium and were then incubated in a humidified atmosphere of 5% CO_2_. Following 4 days of incubation, the culture medium was removed and each well was treated with 10 µL of 3-(4,5-dimethylthiazol-2-yl)-2,5-diphenyl tetrazolium bromide (MTT) (Thermo Fisher Scientific, Waltham, MA, USA), 5 mg/mL in culture medium, and was incubated for 4 hours at 37°C in a humidified atmosphere of 5% CO_2_. The yellow MTT is reduced to blue-purple formazan in the presence of the mitochondrial dehydrogenase. This enzyme is present in intact living cells, hence the blue-purple color produced should be proportional to the number of viable cells present. The MTT solution was then replaced with 100 µL/well of dimethylsulfoxide (DMSO, Paneco Ltd., Moscow, Russia) to dissolve the formazan salts, followed by 10 minutes of slow agitation, yielding a blue-purple solution. The absorbance of this solution was measured at 570 nm using a SPECTROstar® Nano microplate reader for absorbance measurements (BMG LABTECH, Ortenberg, Germany).

Studies of the cellular morphology and attachment were observed using an inverted light microscope Primo Vert (Zeiss, Oberkochen, Germany) and images were captured with a digital camera.

## 3. Results

### 3.1. Mechanical Properties of Chitin–Chitosan Nanocomposites

**Figure 1:**
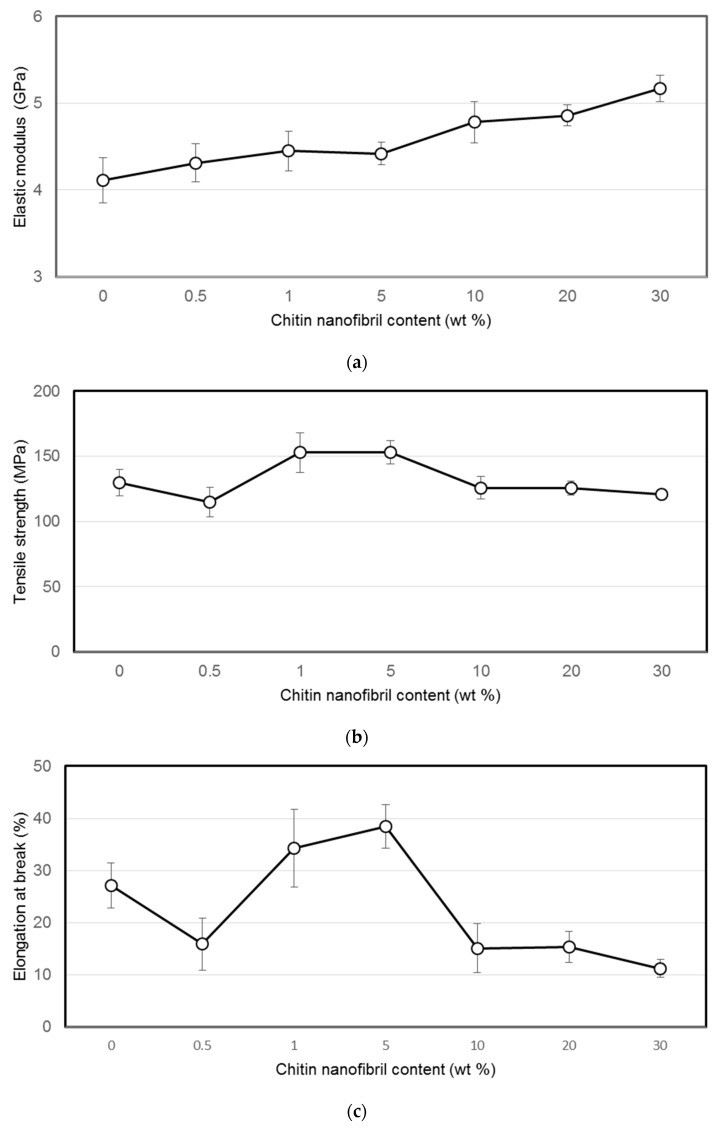
(**a**) Elastic modulus, (**b**) tensile strength, and (**c**) elongation at break of pure chitosan and chitin nanofibril-reinforced chitosan films as a function of chitin nanofibril content.

The mechanical properties of the composite films were determined in a uniaxial tension mode; the elastic modulus (E), tensile strength (σ), and tensile deformation (ε) were measured (Figure 1).

From Figure 1, it follows that the addition of chitin nanofibrils to chitosan films up to 5 wt % with respect to chitosan leads to increases of strength and elongation at break of the composite films, meanwhile the modulus increases constantly with increasing chitin concentration. Tensile strength and elongation at break of chitin nanofibril reinforced chitosan composite films compare well with the results reported by Morganti et al. [12].

Chitin nanofibres are highly compatible with the chitosan matrix [14] due to a large number of hydrogen bonds [11]: chitosan macromolecules contain lateral groups of both NH_2_ and –NH–CO–CH_3_; the latter are characteristic of the chitin macromolecules as well. Hence, higher strength and elastic modulus can be attributed to the additional rigidity from the formation of such favourable interactions. It was also shown [14] that the addition of chitin nanofibrils of more than 1 wt % (with respect to the chitosan), which is the percolation barrier, contributes to the formation of the cluster structure ("the rigid structural network") of chitin nanofibrils. This, in turn, leads to the formation of a more dense macromolecular packaging and a reduction of the chitosan macromolecule mobility and, accordingly, the decrease in strength and elongation at break of composite films when the chitin nanofibril concentration is larger than 1–5 wt %. A steady increase in the module can be explained by the rigidity of the chitin network as well as the high crystallinity of the chitin nanofibrils itself.

### 3.2. Moisture Sorption

The hygroscopic properties of materials characterize their ability to sorb water and water vapour and also to return water to the environment (to desorb). In the case of a high sorptive power of sorbents (polymers) with respect to the sorbate (liquid and its vapour), the sorption process of sorbates by materials leads to an increase in their mass, a change in the structure, and a change in the mechanical properties of the material. This may adversely affect the results of experiments with cell cultures conducted in vitro or in vivo.

Sorption of liquid and its vapours by materials is carried out by adsorption and depends on the composition of the material, the chain flexibility, and chain packing of the polymers. Despite its useful biomedical properties described above, chitosan possesses the high sorptive power due to the presence of hydroxyl, carbonyl, amino, and acetamide groups and, as a result, materials based on it are characterized by instability of mechanical characteristics in the wet state; their strength decreases with an increase of the amount of water sorbed. The addition of hydrophobic chitin nanofibrils to chitosan films should help to reduce their hydrophilicity, which should lead to stabilization of their properties.

**Figure 2:**
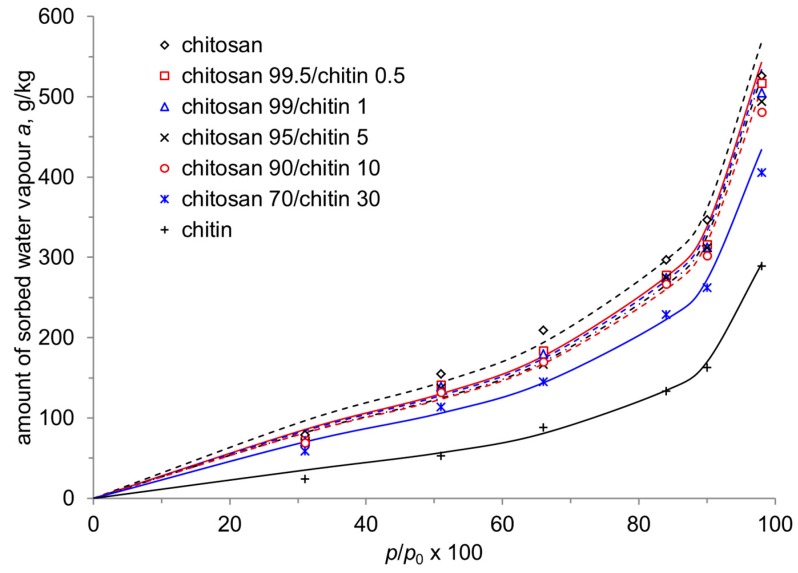
The sorption isotherms of water vapour by chitosan–chitin composite films. The symbols represent experimental data and the lines represent the sorption isotherms calculated by Equation (3).

The sorption isotherms of water vapour by chitosan films and composite films are shown in Figure 2. Sorption isotherms are represented by the dependence of the amount of water vapour sorbed, a, on the relative water vapour pressure, p/p_0_.

The water vapour sorption of the films was described by the thermal equation of sorption

(3) $$a=a_{0}exp\left(-{\left(\frac{-∆\mu_{1}}{E}\right)}^{n}+\alpha \left(T-T_{0}\right)\right),$$

where *a* is the amount of water vapour sorbed at equilibrium water vapour pressure above the sorbent, *p*, and temperature *T*; *a*_0_ is the amount of water vapour sorbed at saturated vapour pressure *p*_0_ = 3.17 kPa and temperature *T*_0_ = 298 K;

(4) $$∆{µ}_{1}=RT\ln\left(\frac{p}{p_{0}}\right)$$

the change in the chemical potential of water vapour during sorption; *Е* is the characteristic energy of sorption; *n* is the Weibull distribution rank (*n* = 0.3–0.33 for densely packed and sorbate-soluble polymers); α is the thermal expansion coefficient and *R* = 8,314 J/mol/K.

The coefficients *a*_0_ and *E* of Equation (3) were calculated from the logarithmic form (5)

(5) $$\ln\left(a\right)=\ln\left(a_{0}\right)-\frac{1}{E^{n}}{\left(-∆{µ}_{1}\right)}^{n}$$

at *Т* = *Т*_0_ = 298 К.

The thermal expansion coefficient α characterizes the main temperature contribution to the sorption value; its sign and value are determined by the interaction energy of water molecules and the elementary unit of the polymer. It can be calculated according to Equation [16]

(6) $$\alpha =1.1\times 10^{-3}-1.12\times 10^{-5}∆E^{*}\left(\pm 9.4\%\right),$$

where $∆E^{*}=\sum ∆E_{i}/M_{i}$ is the specific energy of cohesion (351.7 J/g for chitosan, 385.9 J/g for chitin).

The sorption isotherms shown in Figure 2 are typical for polymeric sorbents swelling in sorbate vapour. Obviously, chitosan films have the highest sorption capacity with respect to water vapour, which may occur due to the presence of polar functional groups and a more labile and less ordered structure of macromolecules. Chitin, characterized by a more ordered structure and high crystallinity [10], has the lowest sorptive power. For composite films, the sorption value decreases with increasing chitin content in the films, although the contributions of the individual components are not additive. This is probably connected with a more dense packing of polymer chains in chitosan–chitin films and the formation of the cluster structure ("the structural network") of chitin nanofibrils. A similar assumption was made by Yudin et al. [14], who showed that the addition of chitin nanofibrils in an amount of more than 1% (percolation barrier) increased the viscosity of composite solutions and prevented the orientational drawing of the fibers during wet spinning.

### 3.3. Water Contact Angle

**Figure 3:**
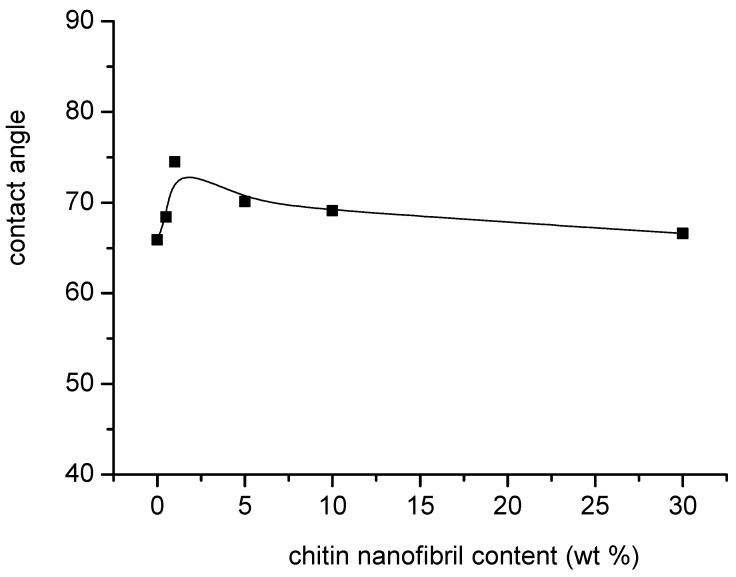
Dependence of the water contact angle on the chitin nanofibrils content of chitosan-based composite films.

Figure 3 shows the dependence of the water contact angle of chitosan-based composite films on the content of chitin nanofibrils. The water contact angle passes a maximum when increasing the chitin nanofibril content from 0% to 5% but remains in the range of 66° to 75°. There is no pronounced dependence of the water contact angle and thus of the wettability of the chitosan-based composite films by water on the content of chitin nanofibrils. Surface hydrophilicity is thus not expected as an important factor to significantly affect cell adhesion and proliferation.

### 3.4. Zeta Potential Analysis

**Figure 4:**
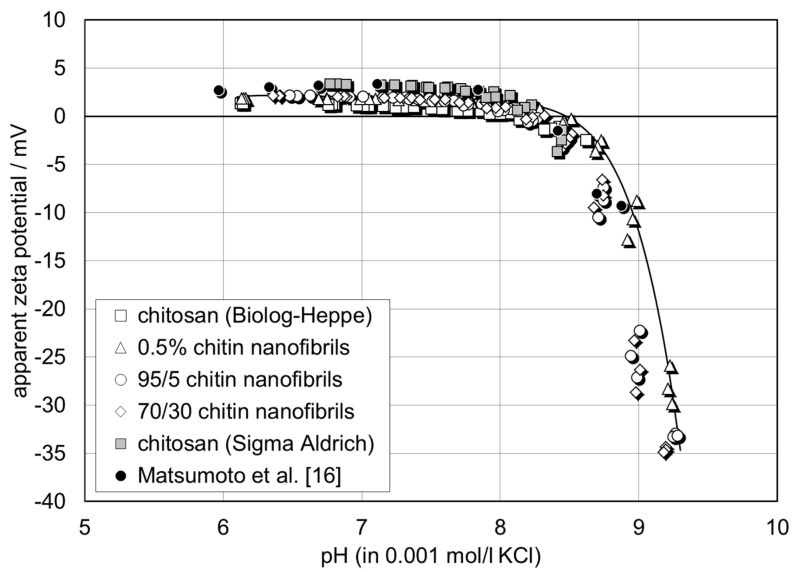
pH dependence of zeta potential for chitin–chitosan nanocomposite films. Comparison with pure chitosan films made of chitosan from different suppliers and with electrospun chitosan nanofiber nonwoven [16].

Figure 4 shows the pH dependence of the zeta potential for a series of chitosan films with 0.5%, 5%, and 30% chitin nanofibrils. The zeta potential determined from the streaming potential measurement is denoted “apparent“ since the contribution of film conductance to the overall conductivity in the capillary channel is neglected in Equation (1). The wet chitin–chitosan nanocomposite films appear to be conductive due to the swelling propensity of chitosan and a possible porosity of the composite films. The apparent zeta potential for the chitin–chitosan nanocomposite films is compared with the zeta potential for pristine chitosan films (without chitin nanofibrils) prepared from two different sources of chitosan: Biolog–Heppe and Sigma Aldrich. At the native pH 6 of the 0.001 mol/l KCl solution, all films show a positive zeta potential in the range of ζ = +1 mV to +7 mV. With increasing pH, the zeta potential remains unchanged until the solution pH approaches the iep of the chitin–chitosan nanocomposite films at pH 8.2 ± 0.1. At pH > iep, the zeta potential assumes a negative sign and decreases rapidly, thereby approaching ζ ~ −30 mV at pH 9. A comparison with the apparent zeta potential and iep reported in the literature shows the same behaviour for a nonwoven film made of electrospun chitosan nanofibers [17]. We conclude that the chitin nanofibrils are fully embedded in the chitosan film matrix and thus do not contribute to the surface charge and zeta potential, respectively.

**Figure 5:**
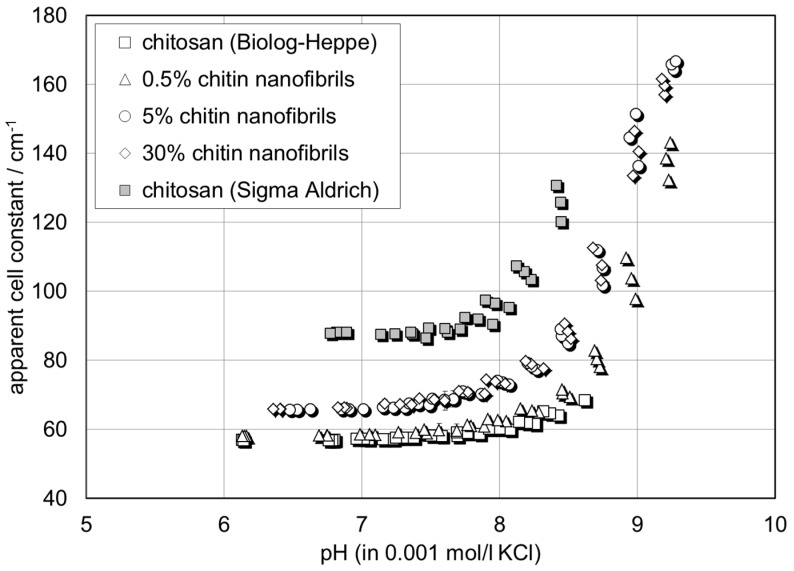
pH dependence of apparent cell constant (Equation (2)) of the rectangular capillary channel for chitin–chitosan nanocomposite films. Comparison with pure chitosan films made of chitosan from different suppliers.

Different to the apparent surface zeta potential, the analysis of the conductance of chitin–chitosan nanocomposite films and its evolution with pH reveals differences in the assumed swelling behaviour of the film samples [18]. Figure 5 shows the dependence of the cell constant calculated from Ohm resistance and bulk electrolyte conductivity measurements according to Equation (2) on the pH of the 0.001 mol/l KCl solution. At a neutral pH, the apparent cell constant assumes *L*/*A*_apparent_ = 60–90 cm^−1^ but increases rapidly above pH ~8, which represents the iep of the chitin–chitosan nanocomposite films. At a higher pH, it approaches the geometric cell constant *L*/*A* = 183 ± 7 cm^−1^, which is expected for a non-porous polymer film that does not show swelling in water. Secondly, the apparent cell constant let us distinguish between three categories of film samples: (a) chitosan (Biolog–Heppe) and chitosan with 0.5% chitin nanofilbrils, which show the smallest apparent cell constant at neutral pH, (b) chitosan with 5% and 30% chitin nanofibrils, respectively, which show a slightly higher apparent cell constant (i.e., a lower conductance of the wet chitosan–chitin nanocomposite films), and (c) the chitosan film made of chitosan obtained from Sigma Aldrich with the highest apparent cell constant at neutral pH. The lower the estimated apparent cell constant, the higher the contribution of electric conductance by the chitin–chitosan nanocomposite films and the higher their swelling propensity. The results for the apparent cell constant correlate qualitatively with the water vapour sorption isotherms in Figure 2. We conclude that the incorporation of chitin nanofibrils in the chitosan matrix suppresses the swelling of the film made of Biolog–Heppe chitosan. At a high pH, swelling is reduced for the complete series of chitin–chitosan nanocomposite films. Swelling is thus caused by the deprotonation of the amine groups of deacetylated chitosan below the iep at pH 8.2 ± 0.1. The pure chitosan film made of chitosan from Sigma Aldrich shows an even lower swelling. This observation correlates with the lower degree of deacetylation for chitosan of Sigma Aldrich (DD 80%) compared to chitosan of Biolog Heppe (DD 92%). Both the incorporated chitin nanofibrils of the chitosan-based composite films and the degree of deacetylation of the chitosan matrix affect the swelling propensity of pure chitosan and composite films with the latter being more significant.

### 3.5. Fibroblast Attachment, Viability, Proliferation, and Morphology

**Figure 6:**
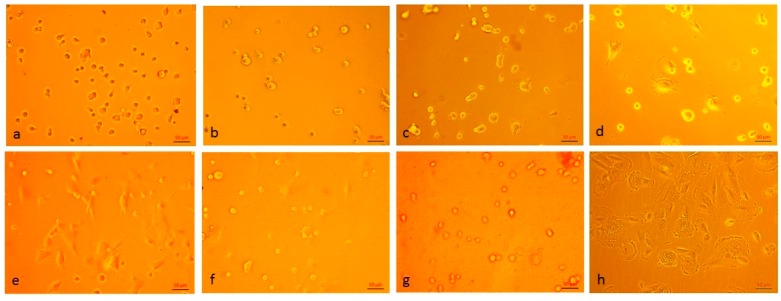
Human dermal fibroblasts were cultured on (**a**) chitosan (CS), (**b**) CS+0.5%CN (chitin), (**c**) CS+1%CN, (**d**) CS+5%CN, (**e**) CS+10%CN, (**f**) CS+20%CN, (**g**) CS+30%CN film matrices, and (**h**) cultural polystyrene surface (control) for 2 hours. Original magnification × 200, phase-contrast light microscopy, scale bar represents 50 μm.

**Figure 7:**
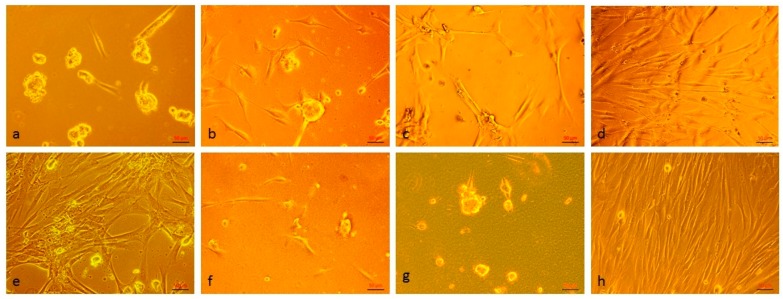
Human dermal fibroblasts were cultured on (**a**) CS, (**b**) CS+0.5%CN, (**c**) CS+1%CN, (**d**) CS+5%CN, (**e**) CS+10%CN, (**f**) CS+20%CN, (**g**) CS+30%CN film matrices, and (**h**) cultural polystyrene surface (control) for 4 days. Original magnification 200X, phase-contrast light microscopy, scale bar represents 50 μm.

**Figure 8:**
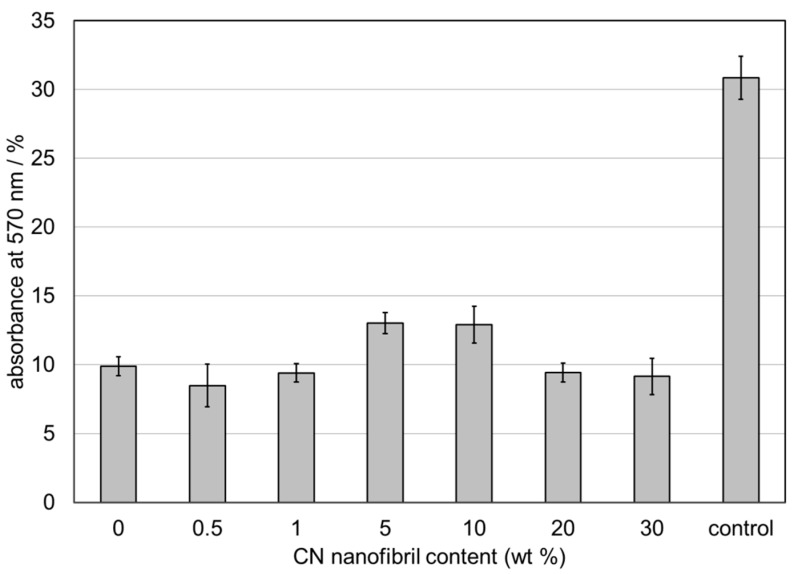
Human dermal fibroblast viability and proliferation on surfaces of film matrices and cultural polystyrene surface (control) as determined by tetrazolium bromide (MTT) assay.

Human dermal fibroblasts were cultured on CS, CS+0.5%CN, CS+1%CN, CS+5%CN, CS+10%CN, CS+20%CN, CS+30%CN film matrices and cultural polystyrene surface (control). Studies of the cellular morphology and attachment were observed using an inverted light microscope and images were captured using a digital camera (Figure 6 and Figure 7). The cell viability and proliferation were evaluated using an MTT test (Figure 8).

After 2 hours of incubation, the effectiveness of the cell adhesion and spreading on CS+10%CN was found to be greater than on other film matrices (Figure 6e). Under the light of the microscope, the development of lamellipodia and filopodia of cells could be observed only in CS+5%CN, CS+10%CN surfaces (Figure 6d,e).

After cell attachment, the cells seeded on CS, CS+0.5%CN, CS+1%CN, CS+5%CN, CS+10%CN, CS+20%CN, CS+30%CN film matrices and cultural polystyrene surface (control) were incubated in culture medium at 37 °C in a humidified 5% CO_2_ incubator for 4 days.

Viable cells on CS+5%CN and CS+10%CN matrices displayed a normal spindle-shaped morphology as well as a spread across the complete surface (Figure 7d,e). The randomly distributed fibroblast cells on the CS+5%CN matrix were connected among each other and formed a cell network across the surface of the scaffold (Figure 7d). The culture on the CS, CS+20%CN, CS+30%CN matrices led to the appearance of small round cells, spherical cell conglomerates, and to considerably elongated rod-like cells (Figure 7a,f,g). The cells on CS+0.5%CN and CS+1%CN matrices were poorly spread and distributed over the surface (Figure 7b,c).

The proliferation rate of cells (for 4 days) on CS+5%CN and CS+10%CN was found to be greater than on CS, CS+0.5%CN, CS+1%CN, CS+20%CN, CS+30%CN film matrices (Figure 8).

The obtained data on the various adhesion efficiencies, the morphological types of cells, the variations in their distribution over the surface of the material, and the nature of the intercellular interactions, as well as the efficiency of proliferation, probably depend on the mechanical properties of the matrix material. Insufficient stiffness of the material is manifested in the form of low adhesion, spreading, and proliferative activity of the cells. Excessive stiffness of the matrix causes priority of the migration activity of cells over proliferative activity.

## 4. Conclusions

Achieving the right balance between substrate mechanics and physicochemical properties in order to achieve specific and well-controlled cellular responses is a challenge. Controlling substrate properties is further complicated by their complex interdependent nature and the fact that these properties are changing spatially and temporally during cellular remodeling. Therefore, in order to generate meaningful design principles for advanced biomaterial surfaces, it is critical to elucidate the manner in which each of these factors act independently, synergistically, or antagonistically to impact cellular behavior. The study of cell behaviors on biomaterials’ surface is of paramount importance because it can reveal many physiological and pathological events and can eventually guide the design of biomaterials with better performance in tissue regeneration.
